# Late-Preterm and Early-Term Respiratory Morbidity in Infants Born Primarily via Elective Caesarean Section

**DOI:** 10.3390/jcm15010126

**Published:** 2025-12-24

**Authors:** Anthoula Arvaniti, Eleni Papachatzi, Emmanuella Magriplis, Nikolaos Antonakopoulos, Leonidas Antonakis, Gabriel Dimitriou, Theodore Dassios

**Affiliations:** 1 Department of Pediatrics, University General Hospital of Patras, 26504 Patras, Greece; med5070@ac.upatras.gr (A.A.); elepapach@upatras.gr (E.P.); gdim@upatras.gr (G.D.); 2 Laboratory of Dietetics and Quality of Life, Agricultural University of Athens, 11855 Athens, Greece; emagriplis@aua.gr; 3 Department of Obstetrics and Gynecology, University General Hospital of Patras, 26504 Patras, Greece; nantonakop@upatras.gr (N.A.);; 4 Neonatal Intensive Care Unit, University General Hospital of Patras, 26504 Patras, Greece

**Keywords:** late preterm, cesarean section, respiratory distress syndrome

## Abstract

**Background/Objectives:** Although morbidity and mortality are more pronounced in extremely and very preterm infants, there is also considerable morbidity in preterm infants of more advanced gestations. Delivery via cesarean section is associated with a higher risk of perinatal complications even when performed electively. Our aim was to examine the possible contribution of prenatal and perinatal factors to the risk for respiratory morbidity in a population of late-preterm and early-term infants delivered with a high rate of elective cesarean section. **Methods:** In a retrospective cohort study, all late-preterm and early-term infants (34 to 38 completed weeks of gestation) that were admitted with respiratory distress to the Neonatal Intensive Care Unit of the University Hospital of Patras over a recent period of two years were included in the study. **Results:** In the study period, 489 infants of all gestations were admitted to the neonatal unit, of whom 221 were born between 34 and 38 + 6 gestational weeks. Ventilated infants had a significantly lower incidence of antenatal corticosteroids (41%) compared to non-ventilated infants (51%, *p* = 0.036) and a higher duration of parenteral nutrition [4 (1–6) days] compared to non-ventilated infants [2 (1–3) days, *p* < 0.001]. The incidence of late-onset sepsis was higher in the ventilated infants (26%) compared to the non-ventilated ones (8%, *p* < 0.001). **Conclusions:** Late preterm and early term infants who were invasively ventilated had less often received antenatal corticosteroids and had a higher incidence of late-onset sepsis compared to those who were not ventilated.

## 1. Introduction

Rather than considering preterm birth as a single homogeneous category, gestational age can be more appropriately regarded as a continuous spectrum with a gradual increase in the risk for morbidities and complications in the lower gestations. Although disease complications and mortality are more pronounced in extremely and very preterm-born infants, there is also considerable morbidity in late-preterm and early-term born infants, which are defined as those born between 34 and less than 39 completed weeks of gestation [1]. Late-preterm and early-term infants constitute a lower risk category compared to very or extremely preterm ones, but they are a larger demographic group and are frequently admitted to neonatal care [2]. Morbidity among late-preterm and early-term infants is more pronounced in the presence of coexisting morbidities and conditions such as pre-eclampsia, intrauterine growth retardation, lack of antenatal corticosteroids, and other perinatal complications [3].

Delivery of term infants via elective cesarean section (CS) is associated with a higher risk for negative perinatal outcomes compared to vaginal delivery, including a higher incidence of respiratory complications such as respiratory distress syndrome and transient tachypnoea of the newborn [4]. Methodologically, the contribution of elective CS in respiratory morbidity can be better studied in a population with a high rate of elective CS, as studies with a lower rate of CS are limited in their ability to account for multiple interrelated prenatal, perinatal, and antenatal confounding factors. The CS rate in countries of the European Union has recently been reported at 26.8% [5], which is considerably lower than the rate reported in most southern European countries, including Greece [6]. The neonatal unit at the University Hospital of Patras (UHP), Greece, serves a diverse community in Western Greece with a high percentage of elective CS. The institution is thus well-placed to delineate the contribution of elective CS in late preterm and early term respiratory morbidity and potentially identify modifiable factors.

Late-preterm infants, accounting for 70–75% of all preterm births [7,8], and early-term infants represent a large demographic group that is often admitted to NICU with increased morbidity [9,10]. There is limited data about the incidence of severe respiratory distress in this population, especially in a setting with an increased incidence of elective cesarean sections, such as Greece. Therefore, it is of great interest to investigate possible related factors that could lead to respiratory morbidity in a population of late-preterm and early-term neonates delivered with elective cesarean section in a tertiary unit (offering the highest national level of NICU services).

The aim of this study was, thus, to examine the contribution of prenatal and perinatal factors to the risk for respiratory morbidity in a population of infants delivered with a high rate of elective CS.

## 2. Materials and Methods

A retrospective cohort study of all admissions to the Neonatal Intensive Care Unit (NICU) of the UHP, Greece, of infants born between 34 and <39 completed weeks of gestation, between 1 January 2023 and 31 December 2024 (two consecutive years), was undertaken. The starting year was selected for purposes of data-entry consistency and to reflect current clinical practice. All data were collected retrospectively during the period between 1 March 2025 and 30 June 2025 by a single researcher who reviewed the Local Unit Database. In case of missing data, medical and nursing notes were reviewed. Data analyses were performed during July 2025.

Eligibility criteria included the following:

- Infants born at 34 to 38 + 6 weeks of gestation
- NICU admission
- Respiratory distress (any)

Exclusion criteria included the following:

- Infants not fulfilling the above criteria
- Infants with congenital anomalies (such as trisomy, congenital heart disease, central nervous system anomaly, etc.)
- Infants with congenital infections

The UHP, Greece, has a tertiary neonatal unit with approximately 1000 deliveries per year and serves a diverse community in western Greece by providing all levels of neonatal care (intensive care, high dependency, and special care). Patras is the third-largest city in Greece and the capital of the 6th Health Administration Authority (ADM, Western Greece). The 6th Health ADM covers an area of 38,350 km^2^, including urban, rural areas, and islands, and covers a population of more than 1.7 million people (16% of the Greek population as per the 2021 Census).

Data for the study were extracted from the Local Unit Database and from medical and nursing notes. The following data were collected: maternal gestational diabetes (yes/no) [11]; maternal hypertension defined as a systolic or diastolic blood pressure ≥ 140 mmHg and ≥90 mmHg, respectively, measured on two occasions 4 h apart (yes/no) [12]; intrauterine growth retardation (yes/no) [13]; maternal age (years); any reported maternal smoking during pregnancy (yes/no); full course of antenatal steroids offered as per protocol before 35 weeks of gestation (yes/no) [14]; mode of delivery (vaginal, elective, or emergency cesarean section); multiple gestation (yes/no); out-born defined as any infant born outside the UHP and postnatally transferred to UHP (yes/no); sex (male/female); gestational age (weeks); birth weight (kg); birth weight z-score; Apgar score at 5 min; Apgar score at 10 min; admission temperature (°C); duration of invasive ventilation (days); duration of non-invasive ventilation (days); duration of supplementary oxygen (days); maximum required fraction of inspired oxygen (FiO_2_); late onset sepsis (yes/no) [10]; duration of antibiotic therapy (days); and duration of parenteral nutrition (PN) (days).

The study was registered with the Department of Clinical Governance of UHP. The study was conducted according to the guidelines of the Declaration of Helsinki and approved by the Hospital’s Ethics Committee of University General Hospital of Patras, (204/13.05.2025).

### 2.1. Ventilation Practice

The infants were ventilated on volume-targeted or pressure-controlled time-cycled ventilation with the Draeger Babylog VN600 neonatal ventilator (Draeger, Lubeck, Germany) or the SLE5000 (SLE, Croydon, UK). Intubation and mechanical ventilation were considered, according to unit policy, when the fraction of inspired oxygen (FiO_2_) was persistently exceeding 0.4, the infant had a pH of <7.25, a partial arterial pressure of CO_2_ of >65 mmHg, or the infant had significant apnoea. Infants were ventilated with a backup rate of 20–40 inflations/min, positive end-expiratory pressure of 4 cm H_2_O, and an inflation time of 0.35 s. All intubated late-preterm and early-term infants with an FiO_2_ requirement of >0.30 and evidence of parenchymal lung disease on chest radiography received endotracheal surfactant [15]. Caffeine was routinely started within the first 6 h of life in infants born before 35 completed weeks of gestation [16].

### 2.2. Statistical Analysis

Continuous data were tested for normality with the Kolmogorov–Smirnov test and found to be non-normally distributed and were therefore presented as median and interquartile range (IQR). The primary outcome was the need for endotracheal intubation and invasive ventilation. As the incidence of invasive ventilation was not different between late-preterm and early-term infants, the study considered both late-preterm and early-term infants as a single group. Binary variables were compared in infants who were invasively ventilated against those who were not, using the Chi-square test. Continuous variables were compared in infants who were invasively ventilated against those who were not, using the Mann–Whitney U non-parametric test. The significance levels are two-sided (Sig.2-tailed), and statistical significance was set at 0.05. Statistical analysis was performed using SPSS software, version 29.0 (SPSS Inc., Chicago, IL, USA).

## 3. Results

### 3.1. Demographics

**Figure 1:**
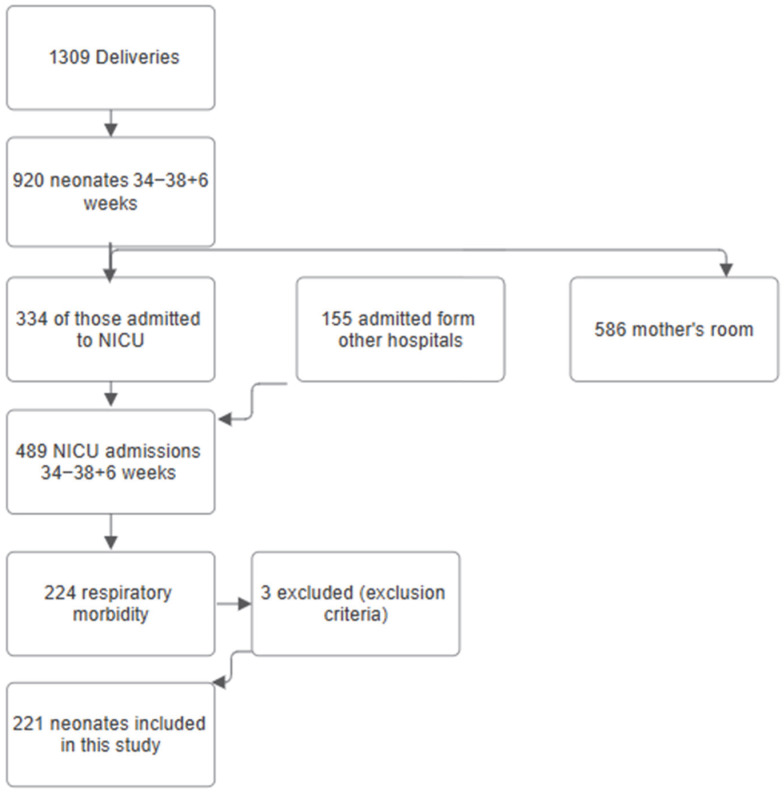
Flow diagram of selection of study population.

During the study period, there were 1309 deliveries at the UHP. Of those, 920 infants were born between 34 and <39 weeks of gestation. In the study period, 489 infants of all gestations were admitted to the Neonatal Intensive Care Unit at the UHP. The rate of admission for inborn infants was 25.5% (excluding 155 neonates that were transferred after delivery). Of the 489 total admissions, 221 were admitted with respiratory morbidity and were born between 34 and <39 weeks (45.1%). Of those, 142 (64.2%) were born between 34 and 36 completed weeks, and 79 (35.7%) between 37 and 38 completed weeks. The selection of the study population is presented in Figure 1. The late-preterm and early-term admitted infants had a median (IQR) gestational age of 35.8 (34.7–37.4) weeks and a birth weight z-score of −0.30 (−1.14–0.78). The prenatal, perinatal demographics and clinical characteristics of the whole population of the admitted late-preterm and early-term infants are presented in Table 1. Prenatal data included maternal diabetes, maternal hypertension, intrauterine growth restriction, maternal age, maternal smoking, and multiple gestation. Perinatal data included antenatal corticosteroid administration, delivery in another hospital (not UHP), cesarean section, elective cesarean section, neonatal sex, birthweight, and Apgar score. Clinical characteristics included temperature on admission, intubation and ventilation, duration (hours) of ventilation, duration (hours) of non-invasive ventilation, duration (hours) of supplementary oxygen administration, maximum required fraction of inspired oxygen (%), duration of antibiotics (days), duration of parenteral nutrition (days), and incidence of early- and late-onset sepsis.

### 3.2. Analyses

#### Comparison of Ventilated and Non-Ventilated Infants

The incidence of invasive ventilation was not significantly different between late-preterm (50 of 142, 35%) and early-term infants (38 of 79, 48%, *p* = 0.064). The ventilated late-preterm and early-term infants did not differ compared to the non-ventilated ones in gestational age (*p* = 0.174), birth weight (*p* = 0.230), and other antenatal and perinatal parameters (Table 2).

The ventilated infants had a significantly lower incidence of antenatal corticosteroids (41%) compared to the non-ventilated infants (51%, *p* = 0.036) and a higher duration of parenteral nutrition [4 (1–6) days] compared to the non-ventilated infants [2 (1–3) days, *p* < 0.001 (Table 2)]. The incidence of late-onset sepsis was higher in the ventilated infants (26%) compared to the non-ventilated ones (8%, *p* < 0.001).

## 4. Discussion

In this study, in a population of late-preterm and early-term infants delivered with a high rate of elective cesarean section, lack of antenatal corticosteroids was associated with a higher incidence of invasive ventilation and the ventilated infants had a more complicated course of stay, as evidenced by a more prolonged course of parenteral nutrition and a higher incidence of late-onset sepsis compared to non-ventilated infants.

The rate of elective cesarean section in our cohort was approximately 65%, which is above the national average. In a study by Kontopanos et al., the rate of cesarean section in the Greek population was reported at 58% [17]. In a recent study covering the period between 1999 and 2023, which presented descriptive data from the majority of European countries, Poland (55.1%), Turkey (54.8%), and Greece (48.6%) had the highest incidence of CS [6]. Delivery via CS is associated with a higher risk of respiratory complications in the offspring (transient tachypnoea, respiratory distress syndrome, and others). The above could be explained by an absence of hormonal signals such as catecholamines, by the lack of stress-related lung maturation, by mechanical aspects (vaginal delivery is thought to exert some beneficial pressure during the passage through the birth canal), and by a delayed transition phase after birth, especially if the CS is planned before 39 weeks of gestation [18,19].

In our cohort, the incidence of respiratory distress reached 40% in late-preterm and early-term infants born via elective cesarean section. Numerous studies have shown that infants delivered via elective CS, especially before 39 weeks of gestation, have higher rates of respiratory complications compared to those born vaginally or after an emergency CS after labor onset [20,21]. More precisely, late-preterm and early-term infants, especially when born via elective cesarean section, are at increased risk for adverse respiratory outcomes compared to those delivered via vaginal delivery [22]. De Luca et al. reported that delivery via elective CS in late-preterm and term infants significantly increased mortality (adjusted risk ratio [aRR]: 2.1), morbidity (NICU admission [aRR: 1.4]), and need for respiratory support (aRR: 1.8) [23]. Late-preterm and early-term neonates had a significantly increased incidence of respiratory distress when delivered by CS [18]. Similar were the findings of Jean-Bernard Gouyon et al., where elective cesarean section was the main risk factor for severe respiratory disorders that required intubation and ventilation in early-term neonates [24]. In a study from Cyprus, male gender and elective CS were identified as independent predictors of respiratory distress in term neonates (OR: 4.35, 95% CI: 1.03–18.39, *p* = 0.045 and OR: 11.92, 95% CI: 1.80–78.95, *p* = 0.01, respectively) [25].

Antenatal corticosteroids administered at gestations before 34 completed weeks are thought to be beneficial for lung maturity in the newborn. According to the American College of Obstetrics and Gynecologists (ACOG), apart from the standard course in preterm deliveries, a single course of betamethasone is recommended for pregnant women between 34 0/7 weeks and 36 6/7 weeks of gestation at risk of preterm birth within 7 days, and who have not received a previous course of antenatal corticosteroids [26]. The British Royal College of Obstetricians and Gynecologists suggests a discussion with the parents regarding antenatal steroids for planned CS in early-term deliveries, focusing on potential risks and benefits [13]. It is uncertain, however, whether antenatal steroids are associated with a reduction in the incidence of respiratory distress syndrome or transient tachypnoea of the newborn, or in the overall rate of neonatal unit admissions. Antenatal corticosteroids may result in some potential harm to the neonate, which includes hypoglycemia and potential developmental delay. The International Federation of Gynecology and Obstetrics (FIGO) highlights that antenatal steroid administration in late-preterm pregnancies reduces the requirement for respiratory support in the first 72 h of life; however, this organization suggests that corticosteroids should not be offered routinely to all women anticipating late-preterm delivery. Instead, prenatal steroid administration should be reserved for women for whom preterm birth is expected within no more than seven days, based on the woman’s symptoms or an accurate predictive test. Some observational studies have reported that antenatal steroids are beneficial in reducing the incidence of respiratory distress in late-preterm and term deliveries [27,28]. In a cost-efficiency analysis using a late-preterm population, antenatal steroid administration was found to decrease healthcare costs and to improve short-term outcomes [29]. On the other hand, antenatal steroids have not been adequately studied in this population (late-preterm, early-term) in the long term. Long-term concerns include neurodevelopmental and metabolic effects and a potential negative impact on growth–immune system regulation. Antenatal corticosteroids have been linked to behavioral and mental disorders in later life [30]. Additionally, they have been related to alterations in glucose metabolism, bone formation, and hypothalamic–pituitary–adrenal axis disturbance, while they might also increase the risk of serious infections during the first 12 months of life [31], and this risk persists until the age of four years [32].

The increased incidence of respiratory disease observed in our cohort could be multifactorial. While the increased rate of elective CS and the lack of antenatal steroids could have been important factors, other causes, such as genetic predisposition, might also play a role. Numerous genetic factors related to respiratory disease have been described, such as mutations in the surfactant metabolism and genetic polymorphisms of the surfactant proteins (SPs) [33,34]. Previous genetic data from our department showed that specific SP-A genetic variants in late-preterm infants may influence susceptibility to RDS independently of the effect of other perinatal factors [35]. To highlight at this point that mechanical ventilation likely reflects the severity of respiratory compromise rather than serving as its causal driver. The observed differences are therefore best interpreted as consequences of prenatal and perinatal lung adaptation failure preceding the need for ventilation, rather than post-intubation ventilatory harm.

Our finding of a higher morbidity in the ventilated infants with more prolonged courses of parenteral nutrition and increased incidence of late-onset sepsis has been previously described in cohorts other than late-preterm and early-term infants [36,37,38]. In the study by Zafar et al., ventilator-dependent infants were at an increased risk of late-onset sepsis [39], while Sharma et al. similarly reported that invasive mechanical ventilation was associated with an increase in the duration of neonatal stay [39,40].

This study has some limitations that are inherent to most retrospective projects. All clinical data consisted of information routinely collected during the clinical course of stay, which resulted in some missing/unknown or incomplete variables. Additionally, authors acknowledge some data heterogeneity, which may have impacted the consistency of our data. Furthermore, some deliveries took place in other centers, and the relevant data were incorporated into the analysis from the respective medical records of the referring hospitals. Similarly, some variables lacked more detailed classification; for example, there was no data regarding the exact extent of diabetes control. Moreover, even though the neonatal unit at UHP serves a diverse community in a large geographical area, the study population was drawn from a single neonatal center, which may reflect specific patient characteristics; therefore, generalizability to other settings might not be guaranteed. In addition, assignment to invasive ventilation cannot be randomized and is determined by clinical necessity for oxygenation and CO_2_ clearance; therefore, causality cannot be inferred. Regarding the observed incidence of CS, it is known that Greece’s CS prevalence has been consistently amongst the highest in Europe [6]. In this study, indications and timing of CS were not assessed. Finally, antenatal corticosteroid courses and timing of administration were not recorded in our cohort.

This study demonstrated that late preterm and early-term neonates delivered via elective CS (mostly without antenatal steroid administration) were intubated and ventilated more often compared to those born vaginally. Moreover, these infants had a prolonged neonatal stay and increased morbidity (longer courses of parenteral nutrition and higher incidence of late-onset sepsis). Our study highlights a significant concern that respiratory morbidity might develop at these advanced gestations, which could be linked to planned elective cesarean and/or lack of antenatal steroids. Our results, although they cannot prove causality (due to methodological design), could potentially imply that elective cesareans should ideally be planned at a full-term gestation to avoid such neonatal complications. The delivery method and timing of delivery are crucial not only to extremely and very preterm infants but also in late-preterm and early-term infants. Antenatal steroids should be offered using an individualized approach, and the risks should be carefully weighed against the benefits in the perinatal period [24]. Population studies are crucial to enhance our understanding of the above parameters in a risk-benefit analysis. Recommendations for future research include larger multicentered studies with long-term follow-up and randomized trials focusing on antenatal steroids (decision to give and timing of administration) in elective cesarean section, to gain a clearer image, which will assist clinicians in the decision-making process regarding antenatal steroid administration, its related risks and benefits, in this specific population (late-preterm and early-term neonates). Additionally, if non-invasive negative pressure ventilation modalities were readily available, future work could independently assess whether intubation and positive-pressure mechanics contribute independently to morbidity.

## 5. Conclusions

In conclusion, late-preterm and early-term infants born with a high rate of elective cesarean section were frequently admitted to neonatal care and had a high incidence of respiratory disease. Late-preterm and early-term infants who were invasively ventilated had less often received antenatal corticosteroids and had a higher incidence of late-onset sepsis compared to late-preterm and early-term infants who were not ventilated.

## Figures and Tables

**Table 1:** Prenatal, perinatal, and clinical characteristics of all admitted late-preterm/early-term newborns (*n* = 221). Data are presented as median (interquartile range, IQR) or *n* (%).

Clinical Characteristics (Prenatal, Perinatal) of Admitted Infants	Median (IQR) or *n* (%)
Maternal diabetes	50 (23)
Maternal hypertension	21 (10)
Intrauterine growth retardation	20 (9)
Maternal age (years)	31 (25–35)
Maternal smoking	12 (5)
Antenatal corticosteroids	104 (47)
Multiple pregnancy	31 (14)
Outborn	86 (39)
Cesarean Section	196 (89)
Elective Cesarean Section	144 (65)
Female sex	79 (36)
Gestational age (weeks)	35.8 (34.7–37.4)
Birth weight (kg)	2.63 (2.25–3.04)
Birth weight (z-score)	−0.30 (−1.14–0.78)
Apgar score at 5 min	9 (9–10)
Apgar score at 10 min	10 (10–10)
Admission temperature (°C)	36.6 (36.3–36.9)
Invasively ventilated	88 (40)
Duration of ventilation (hours)	1 (0–16)
Duration of non-invasive support (hours)	1 (0–6)
Duration of supplemental oxygen (hours)	10 (4–26)
Maximum required fraction of inspired oxygen (%)	40 (30–47)
Duration of antibiotics (days)	5 (3–7)
Duration of parenteral nutrition (days)	3 (2–5)
Early-onset sepsis	8 (3.6)
Late-onset sepsis	33 (15)

**Table 2:** Characteristics of late-preterm/early-term newborns who were invasively ventilated versus those who were not. Data are presented as median (interquartile range, IQR) or *n* (%).

	Ventilated, *n* = 88	Not Ventilated, *n* = 133	*p* Value
Maternal diabetes ^1^	21 (24)	29 (22)	0.869
Maternal hypertension ^1^	12 (14)	9 (7)	0.091
Intrauterine growth retardation ^1^	10 (11)	10 (8)	0.472
Maternal age (years) ^2^	31 (7.5)	31 (10.5)	0.560
Maternal smoking ^1^	5 (6)	7 (5)	1.000
Antenatal corticosteroids ^1^	36 (41)	68 (51)	0.036
Multiple pregnancy ^1^	8 (9)	23 (17)	0.112
Outborn ^1^	36 (41)	50 (38)	0.673
Cesarean Section ^1^	78 (89)	118 (89)	0.642
Female sex ^1^	29 (33)	50 (38)	0.567
Gestational age (weeks) ^2^	35 (2)	36 (2.25)	0.174
Birth weight (kg) ^2^	2.660 (650)	2.620 (930)	0.230
Birth weight (z-score) ^2^	−0.32 (−1.18 to 0.75)	−0.28 (−1.10 to 0.78)	0.685
Apgar score at 5 min ^2^	9 (1)	9 (1)	0.897
Apgar score at 10 min ^2^	10 (1)	10 (1)	0.151
Admission temperature (°C) ^2^	36.6 (0.6)	36.6 (0.5)	0.844
Duration of ventilation (hours) *	19 (12–60)	-	NA
Duration of non-invasive support (hours) ^2^	0 (0–11)	0 (0,0)	<0.001
Duration of supplemental oxygen (hours) ^2^	76 (30–138)	9 (4–23)	<0.001
Maximum required fraction of inspired oxygen (%) ^2^	50 (40–60)	35 (30–40)	<0.001
Duration of antibiotics (days) ^2^	7 (5–7)	3 (3–5)	<0.001
Duration of parenteral nutrition (days) ^2^	4 (1–6)	2 (1–3)	<0.001
Late-onset sepsis ^1^	23 (26)	10 (8)	<0.001

Statistical tests performed are noted by index. ^1^ Pearson’s Chi-square test, ^2^ Mann–Whitney U test, * NA (non-applicable). The significance levels are two-sided (Sig.2-tailed), and statistical significance was set at 0.05.

## Data Availability

Data are available from the corresponding author upon request.

## Abbreviations

The following abbreviations are used in this manuscript:

NICU	Neonatal Intensive Care Unit
UHP	University Hospital of Patras
CS	Cesarean Section
FiO_2_	Fraction of Inspired Oxygen
PN	Parenteral Nutrition
IQR	Interquartile Range
RDS	Respiratory Distress Syndrome
ACOG	American College of Obstetrics and Gynecologists
FIGO	International Federation of Gynecology and Obstetrics
SPs	Surfactant Proteins
NA	Non-applicable
CO_2_	Carbon Dioxide
aRR	adjusted risk ratio
